# Interfacial Dzyaloshinskii-Moriya interaction arising from rare-earth orbital magnetism in insulating magnetic oxides

**DOI:** 10.1038/s41467-020-14924-7

**Published:** 2020-02-27

**Authors:** Lucas Caretta, Ethan Rosenberg, Felix Büttner, Takian Fakhrul, Pierluigi Gargiani, Manuel Valvidares, Zhen Chen, Pooja Reddy, David A. Muller, Caroline A. Ross, Geoffrey S. D. Beach

**Affiliations:** 1 Department of Materials Science and Engineering, Massachusetts Institute of Technology, Cambridge, MA 02139 USA; 2 ALBA Synchrotron Light Source, E-08290, Barcelona, Spain; 3 School of Applied and Engineering Physics, Cornell University, Ithaca, NY 14853 USA

**Keywords:** Magnetic properties and materials, Spintronics, Surfaces, interfaces and thin films

## Abstract

The Dzyaloshinskii-Moriya interaction (DMI) is responsible for exotic chiral and topological magnetic states such as spin spirals and skyrmions. DMI manifests at metallic ferromagnet/heavy-metal interfaces, owing to inversion symmetry breaking and spin-orbit coupling by a heavy metal such as Pt. Moreover, in centrosymmetric magnetic oxides interfaced by Pt, DMI-driven topological spin textures and fast current-driven dynamics have been reported, though the origin of this DMI is unclear. While in metallic systems, spin-orbit coupling arises from a proximate heavy metal, we show that in perpendicularly-magnetized iron garnets, rare-earth orbital magnetism gives rise to an intrinsic spin-orbit coupling generating interfacial DMI at mirror symmetry-breaking interfaces. We show that rare-earth ion substitution and strain engineering can significantly alter the DMI. These results provide critical insights into the origins of chiral magnetism in low-damping magnetic oxides and identify paths toward engineering chiral and topological states in centrosymmetric oxides through rare-earth ion substitution.

## Abstract (web summary)

The origin of interfacial Dzyaloshinskii-Moriya interaction (iDMI) in insulating magnetic oxides remains unclear. Here, Caretta et al. find that an intrinsic spin-orbit coupling due to rare-earth orbital magnetism generates iDMI at mirror symmetry-breaking interfaces of magnetized iron garnets Tm_3_Fe_5_O_12_.

## Introduction

Inversion symmetry breaking gives rise to rich chiral phenomena that yield new fundamental physics and materials properties^1–5^. For example, in chemistry, single-handedness of biomolecules results in specialized functionality during biological operations^2^. In particle physics, a fundamental understanding of electroweak interactions hinges on charge-parity symmetry violations^3^. In magnetic materials, a chiral exchange interaction known as the Dzyaloshinskii–Moriya interaction (DMI) can manifest from broken spatial inversion symmetry^6,7^. This discovery has led to the observation of chiral and topological spin textures, such as magnetic skyrmions and homochiral spin spirals^5,8–13^. Only a limited number of inversion-asymmetric bulk magnetic materials are known, almost all of which exhibit ordered chiral states only at cryogenic temperatures^5,8–11,13^. However, mirror symmetry breaking at interfaces has been shown to give rise to an interfacial DMI (iDMI) in thin-film ferromagnets at room temperature, providing a general route to tailor chirality in magnetic materials through interface engineering^14,15^. iDMI is typically observed in metallic ferromagnet/heavy-metal (HM) bilayers, where strong spin-orbit coupling (SOC) in the HM is responsible for generating the iDMI^14–17^. Since iDMI-inducing HMs such as Pt also give rise to a large spin current from the spin Hall effect^18,19^, topological spin textures in such materials can be efficiently manipulated by electric current^14,15,20^. This has led to large interest in such systems for spintronics applications in which chiral domain walls (DWs) or skyrmions are used to encode bits in racetrack devices^14,15,20–22^.

Very recently, iDMI has been discovered in thin films of centrosymmetric insulating iron garnets^23–25^, an important class of low-damping magnetic oxides in which its presence had gone undetected for decades despite their ubiquity in magnetics research. Experimental observations of homochiral Néel DWs^23,24,26^ and topological Hall-like signals^27,28^ in thin-film rare-earth (RE) iron garnets (REIGs) at room temperature suggest that spin–orbit-driven phenomena thought to be restricted to metallic systems might manifest more broadly in insulating magnetic oxides. However, unlike metallic systems in which iDMI stems from SOC in an adjacent HM, experiments in REIGs suggest that a proximate high-SOC layer is not required. The underlying origin of iDMI in insulating oxides hence remains to be understood.

Here we show that in REIGs, iDMI arises intrinsically at mirror symmetry-breaking interfaces due to SOC in the magnetic oxide itself, owing to the orbital magnetism of the RE ion. We show that the iDMI is influenced by the substrate/oxide and oxide/metal interfaces, but the SOC in the symmetry-breaking layers plays little role in the iDMI. Rather, we show that the iDMI scales with the SOC of the RE ion in the oxide and is undetectable if the RE ion is absent. We further show that the iDMI can be tuned by substrate strain, by at least a factor of two, providing a powerful means to tune chiral magnetism in oxide materials. These results suggest that introducing SOC in magnetic oxides through RE ion substitution can provide a general path toward realizing topological spin states in thin films and heterostructures, opening a new door for oxide-based spintronics.

## Results

### Magnetic and structural characterization

**Fig. 1:**
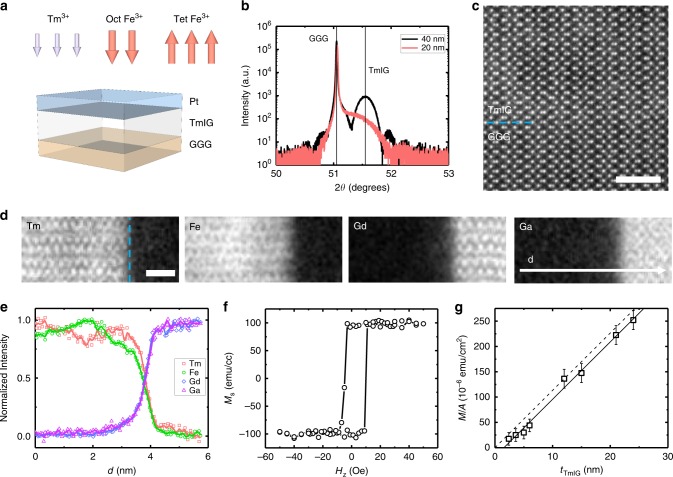
Magnetic and structural characterization of insulating magnetic samples. **a** Schematic of the perpendicularly magnetized GGG/TmIG/Pt layer structure. Red (blue) arrows indicate the orientation of Fe^3+^ (Tm^3+^) sublattice ions in ferrimagnetic TmIG. **b** Omega-2theta (ω−2θ) scans of TmIG films of two different thickness around the GGG(444) substrate peak. **c** High-angle annular dark field (HAADF) scanning transmission electron microscopy (STEM) of the TmIG/GGG interface along the [1${\bar{2}}$1] zone axis, with [111] oriented vertically. Scale bar, 2 nm. **d** Electron energy loss spectroscopy (EELS) maps for metal elements, Tm, Fe, Gd, and Ga. Scale bar, 1 nm. **e** EELS intensity profile across the interface along horizontal direction at distance *d*. **f** Example magnetic hysteresis loop as measured by vibrating sample magnetometry (VSM) of GGG/TmIG (12 nm). **g** Magnetization per unit area (M/A) as a function of TmIG thickness (*t*_TmIG_) as measured by VSM. The error bars represent the noise of the VSM measurement. *M*_s_ saturation magnetization, a.u. arbitrary units. Source data for **g** are provided as a Source Data file.

We first examine the thickness scaling of the iDMI in perpendicularly magnetized Tm_3_Fe_5_O_12_ (TmIG) to verify its interfacial origin and quantify its magnitude. A series of TmIG films were epitaxially grown on (111)-oriented Gd_3_Ga_5_O_12_ (GGG) substrates via pulsed laser deposition (PLD) and covered by a 4.0-nm-thick Pt overlayer (Fig. 1a, see Methods). The nominal TmIG thickness *t*_TmIG_ ranged from 2.4 to 24 nm (approximately 2 unit cells to 20 unit cells). TmIG is ferrimagnetic, with Fe^3+^ ions occupying three tetrahedral and two octahedral lattice sites per formula unit with oppositely oriented magnetic moments. The moment of Tm^3+^ in dodecahedral sites is oriented in the same direction as that of the octahedral Fe^3+^ (Fig. 1a). Figure 1b shows X-ray diffraction performed on exemplary 20 and 40-nm-thick GGG/TmIG samples, revealing *(hhh)*-type reflections. Laue fringes around the TmIG peak of the thicker film indicate the high crystalline quality and thickness uniformity of the magnetic garnet film. Fringes are broader and spaced further apart in the thinner TmIG film. The epitaxial growth of the fully strained garnet film on the GGG substrate is confirmed by reciprocal space mapping^29^ and by high-resolution scanning transmission electron microscopy (Fig. 1c). Complementary electron energy loss spectroscopy (EELS) is shown in Fig. 1d, e, revealing an interface region between the substrate and TmIG of ~1 nm width where Gd and Ga have diffused into the TmIG layer, similar to other studies^30–33^. Perpendicular magnetization in all films is confirmed by vibrating sample magnetometry, shown in Fig. 1f for a 12-nm GGG/TmIG sample. Figure 1g shows the measured magnetic moment per unit area versus *t*_TmIG_, where the fitted slope of the data (solid curve) yields the saturation magnetization (*M*_s_) of the films. We find that TmIG films as thin as 2.4 nm retain near-bulk magnetization with $M_{\mathrm{s}} \approx 115$ emu cm^−3^ beyond an interfacial nonmagnetic layer. The non-zero horizontal intercept indicates a thickness for this layer of *t*_dead_ = 1.4 nm, approximately one unit cell of TmIG, consistent with the STEM and EELS results showing interdiffusion of Fe, Ga, Tm, and Gd at the substrate/film interface. We presume that the non-magnetic dead layer corresponds to the ~1 nm intermixed region at the substrate/garnet interface, where the substitution of Ga for Fe renders the garnet paramagnetic.

### Interfacial DMI in REIGs

**Fig. 2:**
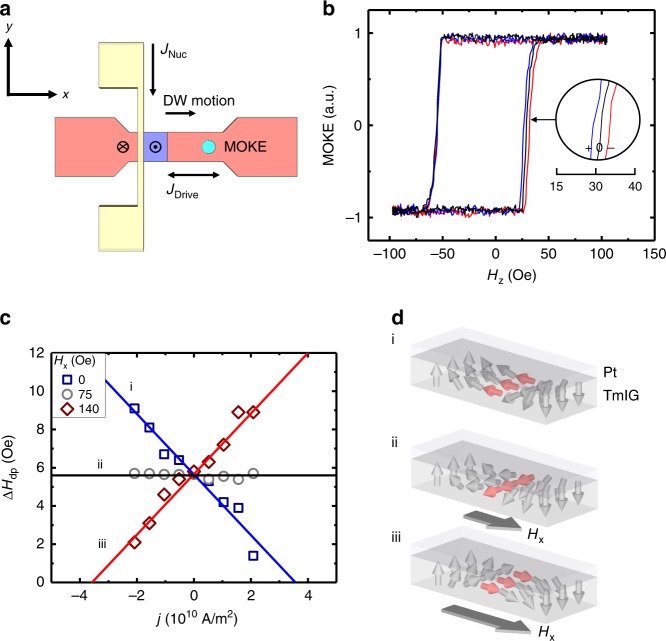
Current-assisted domain wall motion in GGG/TmIG/Pt. **a** Schematic of the domain wall track with electrical connections. Red (blue) regions indicate down (up) net magnetization in the TmIG. Yellow regions represent the domain wall nucleation line. Magneto optical Kerr effect laser spot indicated by a turquoise circle. **b** Exemplary hysteresis loops of GGG/TmIG (6.0 nm)/Pt. Domain wall is nucleated on the positive zero crossing of the magnetic field. Red (blue) loops depict the influence of a positive (negative) current passed through the Pt overlayer. **c** Propagation field (Δ*H*_dp_) as a function of d.c. current density (*J*) for various applied in-plane fields of an up-down domain wall. **d** Schematics illustrating the influence of a longitudinal in-plane field (*H*_*x*_) on a Néel domain wall. Arrow orientation and color indicates the net magnetization. Gray magnetic field arrows indicate the strength of the in-plane field. a.u., arbitrary units.

iDMI was measured using the spin Hall torque magnetometry technique introduced in ref. ^34^, wherein current-induced torque from a spin Hall current injected from the Pt layer is used to probe DW orientation as a function of in-plane applied magnetic field. Samples were lithographically patterned into DW racetracks (Fig. 2a), with Au contacts at either end for current injection. An orthogonal Au strip line was used to nucleate DWs via an Oersted field from a short current pulse (see Methods). DW propagation driven by an out-of-plane field *H*_z_ in the presence of a longitudinal current density *j* was probed using a scanning polar magneto-optical Kerr effect (MOKE) polarimeter (see Methods).

Current flowing in the Pt strip generates a damping-like torque on DWs that acts like an out-of-plane magnetic field $H_{{\mathrm{eff}}} \equiv \chi\, j = \frac{\pi }{2}\chi _0\, j\;{\mathrm{cos}}\left( \psi \right)$, where *χ*_0_ is proportional to the spin Hall angle of Pt (see Methods), and *ψ* is the angle between the DW moment and the current flow direction. *H*_eff_ was extracted from the variation Δ*Η*_dp_ of the DW depinning field under small d.c. current injection (see Methods). The slope of Δ*Η*_dp_ versus *j* (Fig. 2c) yields *χ*, which is finite at zero in-plane field, indicating an equilibrium Néel character to the DW ($|\cos \left( \psi \right)|\, > \, 0$). *χ* changes sign under positive *H*_*x*_ for up-down DWs, as shown in Fig. 2c, d, indicating reversal of the DW chirality from an initially left-handed spiral (leftward-oriented moment). See Supplementary Figs. 3–5 and Supplementary Note 2 for a full explanation of the measurement methodology.

**Fig. 3:**
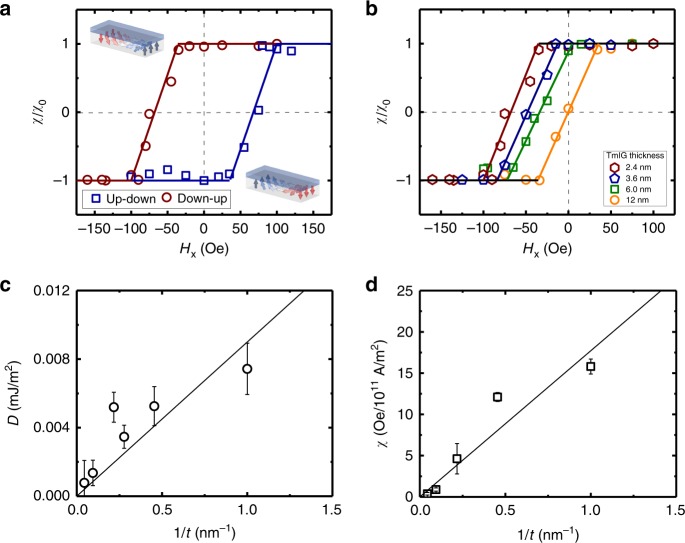
Thickness dependence of the chiral exchange energy in GGG/TmIG/Pt. **a** Normalized spin Hall efficiency (*χ*/*χ*_0_) as a function of in-plane field (*H*_*x*_) for up-down (open blue squares) and down-up (open red circles) domain walls. Insets are schematics of up-down and down-up domain walls at zero applied field. **b** *χ*/*χ*_0_ for down-up domain walls in various thicknesses of GGG/TmIG/Pt films. TmIG thickness (*t*) dependence of the **c** Dzyaloshinskii–Moriya interaction energy (*D*) and **d** the spin Hall efficiency. Error bars in **c** are the propagated error of measured *M*_s_, Δ, and *H*_*D*_ at each thickness, while the error bars in **d** are the standard error of 4–6 measurements at each thickness. Source data for **c** and **d** are provided as a Source Data file.

Figure 3a shows $\chi /\chi _0 \propto \cos \left( \psi \right)$ versus *H*_*x*_ for up-down and down-up DWs in GGG/TmIG (2.4 nm)/Pt (4.0 nm). The data indicate that both DW types are fully Néel at *H*_*x*_ = 0, with oppositely oriented moments and hence with the same left-handed chirality, in agreement with other work^23,24^. The curves in Fig. 3a are analogous to shifted hard-axis hysteresis loops of the DWs: the field required to rotate the DW moment from Néel (*χ* = ±1) to Bloch (*χ* = 0) is related to the DW shape anisotropy field *H*_*k*_, and the zero-crossing of the loops corresponds to the DMI effective field *H*_D_ that biases the DW in a Néel configuration. The solid lines in Fig. 3a are a fits of cos(*ψ*) versus *H*_*x*_ using a simple 1D DW energetics model, yielding *H*_*k*_ = 80 Oe and *H*_*D*_ = 70 Oe (see Methods).

Figure 3b shows similar results for down-up DWs in films with varying *t*_TmIG_, from which we find that *H*_*D*_ decreases with increasing *t*_TmIG_. We computed the DMI energy density $D = \mu _0H_DM_{\mathrm{s}}{\mathrm{\Delta }}$ using the DW width Δ obtained from ${\mathrm{\Delta }} = M_{\mathrm{s}}t_{{\mathrm{TmIG}}}\ln (2)/\pi H_{{k}}$^35^. Figure 3c shows that *D* scales as $\tilde D/t$, where $t = (t_{{\mathrm{TmIG}}} - t_{{\mathrm{dead}}})$ is the effective magnetic layer thickness and $\tilde D$ is the interfacial DMI energy density. The 1/*t* dependence of the data confirms the interfacial origin of the DMI, with $\tilde D = 8.99 \times 10^{ - 12}\,{\mathrm{mJ}}/{\mathrm{m}}$. We also find that *χ* scales inversely with *t*, implying a constant effective spin Hall angle *θ*_eff_~0.5% (see Methods). Together with the data in Fig. 1c, this indicates the absence of dimensional crossover effects at small *t*_TmIG_ in our films, in contrast to the conclusions drawn from the data presented in ref. ^30^.

**Fig. 4:**
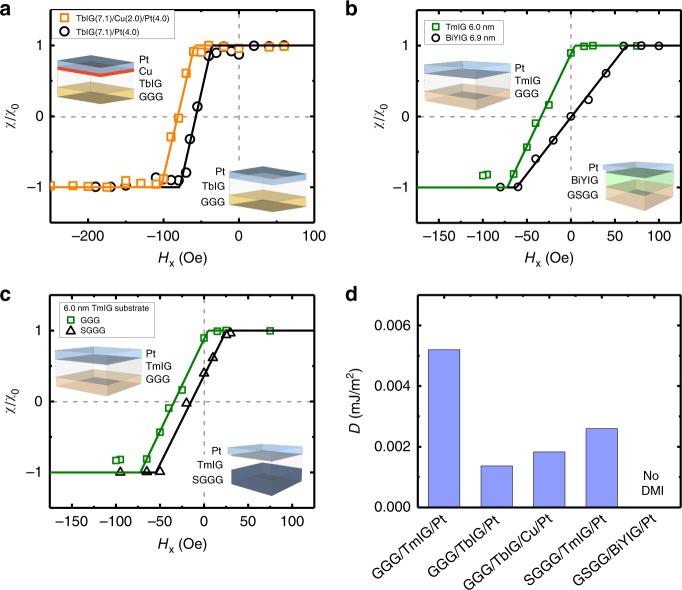
In-plane field dependence of the spin Hall efficiency in Pt-covered insulating magnetic garnets. Normalized spin Hall efficiency (*χ*/*χ*_0_) performed on up-down domain walls in **a** GGG/TbIG (7.1 nm)/Pt (4.0 nm) and GGG/TbIG (7.1 nm)/Cu (2.0 nm)/Pt (4.0 nm), **b** GGG/TmIG (6.0 nm)/Pt (4.0 nm) and GSGG/BiYIG (6.9 nm)/Pt (4.0 nm), and **c** GGG/TmIG (6.0 nm)/Pt (4.0 nm) and SGGG/TmIG (6.0 nm)/Pt (4.0 nm). Insets are layer schematics of each thin film system. **d** Summary of DMI (*D*) strengths in each system in **a**–**c**.

As shown previously^23,24^, the DW chirality is opposite to that expected if the Pt interface were the source, since here the DWs are left-handed despite Pt being on top. This suggests that the iDMI in garnets has a different origin than in metallic ferromagnet/HM systems, since the SOC in Pt would be expected to generate right-handed chirality in the present layer geometry. Figure 4a shows spin Hall torque magnetometry data for TbIG (7.1 nm)/Pt (4.0 nm) and TbIG (7.1 nm)/Cu (2.0 nm)/Pt (4.0 nm) films, which show the influence of the RE ion and of the adjacent HM on the iDMI. Both films exhibit sizable iDMI, but when accounting for *M*_s_ and *D*, we find *D* = 0.0013 mJ/m^2^ for TbIG (7.1 nm)/Pt (4.0 nm), approximately four times smaller than for TmIG/Pt of similar thickness. Hence, changing the RE ion results in a substantial change in *D*. By contrast, inserting a thin Cu spacer only slightly changes the iDMI. Hence, the relative difference between having a strong SOC metal in direct contact with the garnet or not is small compared to the effect of changing the RE ion in the oxide itself. Indeed, the iDMI actually increases slightly upon Cu layer insertion, to *D* = 0.0018 mJ/m^2^, which suggests that symmetry breaking at the oxide/metal interface plays a role in the iDMI, but that the strength of the SOC in the metal at this interface does not play a significant role. The fact that the magnitude of the iDMI changes only weakly when changing the interfacing metal, whereas it varies strongly with changes in the RE orbital moment, indicates that it is the SOC of the RE ion at mirror symmetry-breaking interfaces that primarily determines the iDMI strength.

### Role of rare-earth orbital magnetism in DMI

To verify the role of the RE ion in generating iDMI, we perform similar measurements using perpendicularly magnetized Bi-substituted yttrium iron garnet (BiYIG, see Methods) that lacks a RE ion. Here Bi and Y ions occupy the dodecahedral sites, with Bi yielding PMA when the film is under in-plane tensile strain^36^. We examined a GSGG/BiYIG (6.9 nm)/Pt (4.0 nm) film, where Sc-doped GGG (GSGG, lattice parameter 12.554 Å) was used as the substrate to achieve the necessary tensile strain state in BiYIG (see Supplementary Figs. 1 and 2 and Supplementary Note 1 for structural characterization). We find no detectable influence of current on DW motion at *H*_*x*_ = 0 in this case, indicating purely Bloch DWs at equilibrium. At sufficiently large $|H_x|$, current-assisted motion with a spin Hall torque efficiency *χ* = 13.8 Oe per 10^11^ A/m^2^ is observed, indicating that the lack of current-induced effects at zero *H*_*x*_ is due to a lack of DMI. This can be seen from the spin Hall torque magnetometry data in Fig. 4b. The fact that no detectable DMI is observed in this film, even though Pt is in direct contact, strongly suggests that the SOC in the metal layer plays no role in generating iDMI in these materials. We note that the STEM and EELS results indicate elemental diffusion at the substrate/garnet interfaces, consistent with the formation of a dead layer, and this could contribute to inversion symmetry breaking, and hence to DMI. However, the lack of measurable DMI in the BiYIG sample suggests that the contribution by such interdiffusion gradients is very small compared to the DMI that emerges from the presence of RE ion in the garnet.

If the orbital moment of the RE ion is responsible for the iDMI, then one should expect that the iDMI would be sensitive to the garnet strain state, similar to the magnetic anisotropy, which originates from magnetoelastic coupling mediated by the RE ion. Figure 4c compares spin Hall torque magnetometry data for TmIG (6.0 nm)/Pt (4.0 nm) films grown on GGG and on substituted-GGG (SGGG), with lattice constants $a_{{\mathrm{GGG}}} = 12.376{\mathrm{{\AA}}}$ and $a_{{\mathrm{SGGG}}} = 12.480{\mathrm{{\AA}}}$, respectively. We find *D* = 0.0026 mJ/m^2^ for the film grown on SGGG, half the value that it takes in the less-strained film on GGG, which shows the clear influence of strain on the iDMI. A summary of these results is shown as a bar chart in Fig. 4d.

**Fig. 5:**
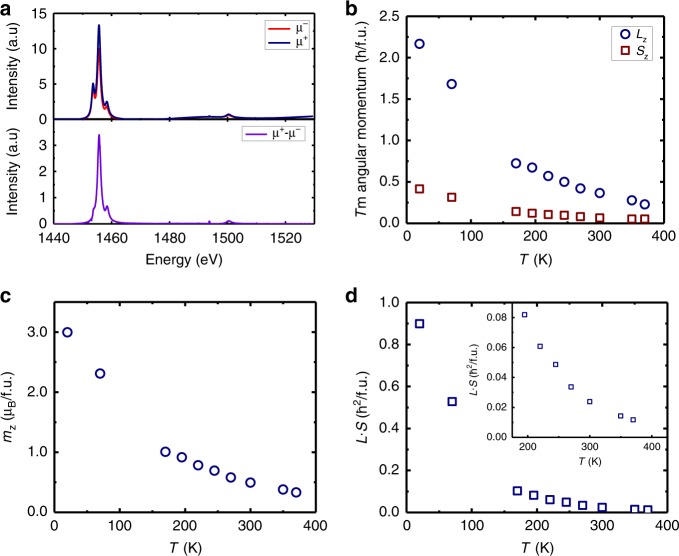
X-ray magnetic circular dichroism on GGG/TmIG (6.0 nm). **a** Exemplary Tm XMCD spectrum under 10 kOe field at *T* = 70 K showing positive (*μ*^+^) and negative (*μ*^−^) helicity and a difference spectrum (*μ*^+^–*μ*^−^). *z*-component of the **b** orbital (*L*_*z*_) and spin (*S*_*z*_) angular momentum and **c** magnetization **m**_***z***_ as a function of temperature (*T*). **d** Temperature dependence of spin–orbit coupling (${\mathbf{L}} \cdot {\mathbf{S}}$). The inset in **d** expands the data between 200 and 400 K for clarity. The error bars in **b**–**d** are smaller than the data points and represent standard errors estimated from a variation of the sum rule integration boundaries. In addition, there is a systematic measurement uncertainty of 20% on the absolute scale. a.u., arbitrary units. f.u., formula unit.

DMI requires both broken inversion symmetry and strong SOC. In thin film heterostructures, an HM layer typically provides both of these ingredients. However, the results above suggest that in the RE garnets, the RE ion in the magnetic film itself provides the critical SOC responsible for DMI, irrespective of the SOC of the interfacing material. Although RE ions exist in the bulk of the garnet, they only contribute to DMI at film surfaces and interfaces where the requisite symmetry breaking exists. The SOC Hamiltonian is given by $\widehat {H_{{\mathrm{SO}}}} \propto {\mathbf{L}} \cdot {\mathbf{S}}$, where **L** and **S** are the orbital and spin angular momentum, respectively. We used element-specific X-ray magnetic circular dichroism (XMCD) to directly measure both **L** and **S** in TmIG to explicitly determine the contribution of the RE ions to the SOC, and thus to the iDMI. Figure 5a shows exemplary XMCD spectra for Tm^3+^ in GGG/TmIG(6.0 nm) without Pt cap for both positive (*μ*^+^) and negative (*μ*^−^) X-ray helicity, as well as the XMCD difference (*μ*^+^–*μ*^*−*^) along the *z*-axis. Using dichroism sum rules (see Methods), we separately find the orbital *L*_*z*_ and spin *S*_*z*_ contributions to the angular momentum from the integrated XMCD signal, which are plotted in Fig. 5b as a function of temperature (*T*). From these data, we computed the Tm^3+^ moment per formula unit, $m_z = - \mu _{\mathrm{B}}L_z - 2\mu _{\mathrm{B}}S_z$ (Fig. 5c), and the normalized SOC ${\mathbf{L}} \cdot {\mathbf{S}}$ ($\propto \widehat {H_{{\mathrm{SO}}}}$) per formula unit (Fig. 5d) as functions of *T*. As **L** is quenched in Fe^3+^, it is not expected to contribute to the SOC in TmIG, whereas the SOC from Tm^3+^ is significant and strongly *T*-dependent. An XMCD-measured Gd hysteresis loop performed on the GGG/TmIG(2.4 nm) sample is shown in the Supplementary Fig. 6 and described in Supplementary Note 3, illustrating Gd paramagnetism. Thus, we can confirm that substrate Gd and any Gd that diffused into the interfacial dead layer is not ferrimagnetic at room temperature.

**Fig. 6:**
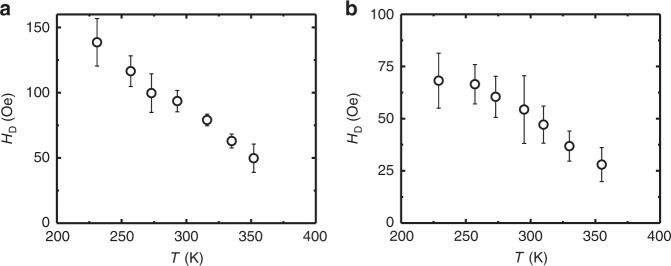
Temperature dependence of DMI. The DMI effective field (*H*_*D*_) as a function of temperature (*T*) in **a** GGG/TmIG (2.4 nm)/Pt (4.0 nm) and **b** GGG/TmIG (6.0 nm)/Pt (4.0 nm). Error bars are standard error of three measurements. Source data are provided as a Source Data file.

If the RE-mediated SOC is responsible for the iDMI in these materials, one would expect a similarly strong dependence of the iDMI on *T*. To confirm this, we measured the DMI effective field *H*_D_ in GGG/TmIG(*t*_TmIG_)/Pt (4.0 nm) as a function of *T*, as shown in Fig. 6a, b for *t*_TmIG_ = 2.4 and 6.0 nm, respectively. Similar to the SOC in Tm^3+^, we find that *H*_D_ in both samples scales nearly linearly with *T* in the measured range, providing further evidence that the iDMI in REIG films originates from SOC coupling in the RE ion.

## Discussion

In summary, we examined the chiral Dzyaloshinskii–Moriya exchange interaction in ultrathin centrosymmetric insulating magnetic garnets. We find that the origin of the DMI is interfacial, and that in substrate/REIG/metal trilayers, both the substrate and the metal overlayers contribute to the strength of the DMI. Nevertheless, our experiments cannot separate the iDMI contribution from the top and bottom interfaces, since we only measure a single, net iDMI. However, in contrast to metallic heavy-metal/ferromagnet heterostructures, in which the SOC in the heavy metal plays a critical role, in magnetic garnets, it is the SOC in the oxide itself, arising from RE orbital magnetism, that is responsible for the iDMI. We find that the iDMI can be tuned with substrate strain, and that in TmIG it is strongly temperature dependent, tending to vanish at about 350 K. Intriguingly, we find that the Tm^3+^ SOC increases by about a factor of 40 at low temperature compared to room temperature, which suggests that the iDMI at low T may likewise show a giant enhancement. While these results may give pause to the interpretation of anomalies in the Hall effect signals at elevated temperatures as resulting from chiral magnetic states, they may point to chiral phases such as spontaneous skyrmion lattices that could emerge at low *T* where iDMI is expected to be substantial. These results also provide critical insights that could allow the iDMI to be substantially enhanced, by engineering the RE content and SOC in garnets, and perhaps in magnetic oxides generally. Our observations hence point to new avenues and opportunities to engineer chiral magnetism in magnetic oxides through RE ion substitution.

## Methods

### Growth, characterization, and patterning of materials

TmIG and TbIG films were deposited using PLD on single-side-polished, single-crystal GGG and SGGG substrates following a previously reported method^37^. The PLD used a 248 nm wavelength KrF excimer laser with 10 Hz repetition rate and a heated substrate stage. The target used was a commercially available TmIG target with a 99.9% elemental purity. The target–substrate distance was fixed at 8 cm. Epitaxial growth of the films was confirmed using X-ray diffraction 2*θ*–*ω* scan of the (444) reflection. Film thickness was determined by X-ray reflectometry. Atomic force microscopy RMS roughness measurements were carried out in a Digital Instruments Nanoscope IV with a 1 μm × 1 μm scan size and XRD measurements were carried out in a Bruker D8 Discover HRXRD. Tapping-mode AFM of the 2.4-nm-thick TmIG sample used in this study gave an RMS roughness of 0.64 nm. Cross-sectional samples for scanning transmission electron microscopy (STEM) were prepared using Ga^+^ ion milling by a focused ion beam (Thermo Fisher Strata 400). The ion beam was reduced to 2 keV for the final thinning step to reduce sample damage. STEM experiments were carried out using a probe aberration corrected microscope (Thermo Fisher Titan Themis) operating at 300 keV and with a probe-forming semi-angle of 21.4 mrad.

The BiYIG (6.9 nm) film was deposited by PLD on single-side-polished, single crystal Gd_3_Sc_2_Ga_3_O_12_ (111) substrates using a 248 nm KrF excimer laser of fluence ~2 J/cm^2^ and a laser repetition rate of 10 Hz^38^. The stoichiometric Bi_0.8_Y_2.2_Fe_5_O_12_ target was prepared from Fe_2_O_3_ and Bi_2_O_3_ powder by a mixed oxide sintering method. The chamber was pumped to 5 × 10^−6^ Torr base pressure prior to introducing oxygen and depositing the films. The target–substrate distance was fixed at 6 cm. During deposition the substrate temperature was 560 °C and the oxygen pressure was 100 mTorr. The films were cooled to room temperature at 10 °C min^−1^ and 225 Torr oxygen pressure. High-resolution X-ray diffraction (HRXRD) 2*θ*–*ω* scans of the (444) reflection reveal the epitaxial growth of the films and film thicknesses were determined by X-ray reflectometry.

The perpendicular easy axis of the REIG films is due to magnetoelastic anisotropy arising from epitaxial growth on the GGG and SGGG substrates. TmIG is under in-plane tensile strain with a negative magnetostriction coefficient *λ*_111_ (ref. ^29^); TbIG is under in-plane compression with a positive *λ*_111_ (ref. ^37^). Replacing Y with Bi in dodecahedral sites of BiYIG in sufficient quantities expands the lattice parameter and increases the magnitude of the negative magnetostriction coefficient; growth on substituted garnet substrates leads to tensile strain yielding an out-of-plane magnetic easy axis^36^.

Cu and Pt metallic overlayers were grown using d.c. magnetron sputtering with an Ar sputter gas pressure of 3 and 3.5 mTorr, respectively, and a background base pressure of 1 × 10^−7^ Torr. Deposition rates were calibrated using X-ray reflectivity measurements. DW motion tracks were patterned using standard photolithography and ion milling. The contact pads (Ta (6 nm)/Au (150 nm)) were patterned using photolithography and lift-off processes. Spin Hall Torque magnetometry measurements were performed on 50 μm × 40 μm tracks.

### Magneto-optical Kerr effect (MOKE)

Polar MOKE measurements were acquired on a custom-built, three-axis scanning Kerr microscope with independent out-of-plane and in-plane magnetic field control. The light source was a continuous-wave 445 nm diode laser focused with a ×10 objective to a spot size of ~8 μm. The laser was attenuated to ~3 mW to prevent heating on the sample. Temperature was controlled via an integrated flow cryostat.

### Spin Hall torque magnetometry

To characterize the effective field *H*_eff_ from the spin current generated by the Pt overlayer, we followed ref. ^34^ and measured the depinning field *H*_dp_ of a DW under a d.c. bias current in lithographically defined DW racetracks. The black curve in Fig. 2b shows a polar MOKE hysteresis loop for GGG/TmIG (6.0 nm)/Pt (4.0 nm). During the field sweep, a DW is nucleated by passing a short current pulse through the overlaid Au stripline during the zero-field crossing of the positive field sweep. As the field ramps, the DW propagates towards the focused MOKE laser spot (Fig. 2a). This results in an asymmetric hysteresis loop, where the negative switching field corresponds to the DW nucleation field of the sample *H*_nuc_, and the positive switching field is the magnetic field required to depin an already nucleated DW *H*_dp_. To measure the effect of the spin–orbit torque from the Pt overlayer, a current is injected concurrently with the propagating DW to help or hinder its motion. This results in a decrease or increase in *H*_dp_ (Fig. 2b blue and red curves, respectively). The change in propagation field Δ*H*_dp_ with in-plane field is the spin Hall efficiency *χ*.

We fit the spin Hall torque magnetometry data to $\chi = \frac{\pi }{2}\chi _0\cos \left( \psi \right)$, where *ψ* is the angle between the DW moment and the *x-*axis. *ψ* is computed as a function of *H*_*x*_ and *H*_*y*_ using a 1D DW model with DMI^39^, where the DW surface energy density *σ* is written as

$$\frac{\sigma }{{2\Delta \mu _0M_{\mathrm{s}}}} = \frac{1}{2}H_{{k}}{\mathrm{cos}}^2\left( \psi \right) - \frac{\pi }{2}H_{{D}}{\mathrm{cos}}\left( \psi \right) - \frac{\pi }{2}H_x{\mathrm{cos}}\left( {\uppsi} \right) - \frac{\pi }{2}H_y{\mathrm{sin}}\left( \psi \right) + H_ \bot,$$

where $H_ \bot$ is the perpendicular anisotropy field. Minimizing *σ* with respect to *ψ* yields an expression that relates cos(*ψ*) ∝ *χ* to an applied in-plane field *H*_*x*_ and *H*_*y*_. Thus, *χ* can be used as a probe of the in-plane component of the DW magnetization. For this case of *H*_*x*_,

$$cos(\psi) = \left\{ \begin{array}{*{20}{cc}} + 1 & \left( {H_{{D}} + H_x} \right)\; > \; \frac{2}{\pi }H_{{k}} \hfill\\ \pi \left( {H_{{D}} + H_x} \right)/2H_k & - \frac{2}{\pi }H_k\; <\; \left( {H_{{D}} + H_x} \right) < \frac{2}{\pi }H_{{k}} \\ - 1 & \left( {H_{{D}} + H_x} \right)\; < - \frac{2}{\pi }H_{{k}} \hfill\end{array} \right.$$

### Calculation of spin Hall angle *θ*_eff_

A current passing through the Pt overlayer acts as an easy-axis effective field $H_{{\mathrm{eff}}} \equiv \chi j = \frac{\pi }{2}\chi _0j\;{\mathrm{cos}}\left( \psi \right)$. Here $\chi = \frac{\pi }{2}\frac{{\hbar {\uptheta}_{{\mathrm{eff}}}}}{{2e\mu _0M_{\mathrm{s}}t}}$, where *θ*_eff_ is the effective spin Hall angle, *e* is the electron charge, *M*_s_ is the saturation magnetization, and *t* is the magnetic film thickness. From Fig. 1c, we find a constant *M*_s_ with TmIG thickness. Using the linear fit to the *χ* versus 1/*t* data in Fig. 3d, we calculate *θ*_eff_ ~ 0.5%.

### X-ray magnetic circular dichroism

The Tm XMCD measurements (experiments) were carried out at the BOREAS beamline of the ALBA synchrotron using 90% circularly polarized X-ray beam produced by an Apple-II type undulator^40^. The base pressure during measurements was <7 × 10^−11^ mbar. The X-ray beam was focused to about 500 μm × 500 μm and a gold mesh was used for incident flux signal normalization. The X-ray absorption signal was measured with a Keithley 428 current amplifier via the sample-to-ground drain current (total electron yield TEY signal). All measurements were conducted in a magnetic field of 1 T generated collinearly with the incoming X-ray direction by a superconducting vector-cryomagnet (Scientific Magnetics). This field strength was chosen to align the net magnetization without causing spin-flop.

Two spectra per helicity were recorded across the Tm *M*_4_ and *M*_5_ edges at normal incidence. Such XMCD measurements provide information to extract the spin and angular moment along the beam direction (*S*_*z*_ and *L*_*z*_, respectively). The procedure is described in detail in refs. ^41,42^. We first multiplied each spectrum with a constant factor such that the average intensity in the pre- and post-edge regions of all spectra aligned. We manually discard spectra with intensity drift, i.e., spectra where such alignment was not possible. The sum and the difference of the remaining alternating helicity spectra were calculated to obtain the XMCD and the XAS spectra, respectively. XAS spectra were further background-corrected by subtracting a linear interpolation in the off-peak intervals *E* < 1445 eV, 1465 eV < *E* < 1498eV, and *E* > 1504 eV. We then define *p* as the integral of the XMCD over the *M*_5_ peak ($p = \smallint_{M_5} {\mathrm{XMCD}}\;{\mathrm{d}}E$), *q* as the total XMCD integral ($q = \smallint _{M_4 + M_5} {\mathrm{XMCD}}\;{\mathrm{d}}E$), and *r* as the total integral over the background-corrected XAS ($r = \smallint_{M_4 + M_5} {\mathrm{XAS}}\;{\mathrm{d}}E$). Error bars of *p* and *q* were derived by variation of the actual integration limits. The magnetic moments per formula unit were then calculated as $L_z = 2\hbar n_hq/r$ and $S_{{\mathrm{eff}}} = \hbar n_h(5p - 3q)/r$, where *n*_*h*_ = 6 is the number of empty 4*f* states of Tm per formula unit (2 per ion) and *S*_eff_ = 2*S*_*z*_ + 6*T*_*z*_ is the effective spin density that includes the magnetic dipole moment *T*_*z*_^42^. Assuming that the ratio of *S*_*z*_/*T*_*z*_ is constant, we have used literature values for the free Tm ion $S_z^{{\mathrm{free}}}/T_z^{{\mathrm{free}}} = ( - 0.991)/( - 0.407)$^43^ to calculate *S*_*z*_ from the measured *S*_eff_ as $S_z = S_{{\mathrm{eff}}}/(2 + T_z^{{\mathrm{free}}}/S_z^{{\mathrm{free}}})$^42^.

## Supplementary information

Supplementary Information

## Data Availability

The data that support the findings of this study are available from the corresponding author upon reasonable request. The source data underlying Fig. 1g, 3c, d, 6 are provided as a Source Data file.

## Source data

Source Data

## References

[1] Anderson PW (1972). More is different. Science.

[2] Siegel JS (2001). Single-handed cooperation. Nature.

[3] Ellis J (2003). Antimatter matters. Nature.

[4] Bode M (2007). Chiral magnetic order at surfaces driven by inversion asymmetry. Nature.

[5] Yu XZ (2010). Real-space observation of a two-dimensional skyrmion crystal. Nature.

[6] Dzyaloshinsky I (1958). A thermodynamic theory of “weak” ferromagnetism of antiferromagnetics. J. Phys. Chem. Solids.

[7] Moriya T (1960). Anisotropic superexchange interaction and weak ferromagnetism. Phys. Rev..

[8] Mühlbauer S (2009). Skyrmion lattice in a chiral magnet. Science.

[9] Yu XZ (2011). Near room-temperature formation of a skyrmion crystal in thin-films of the helimagnet FeGe. Nat. Mater..

[10] Rößler UK, Bogdanov AN, Pfleiderer C (2006). Spontaneous skyrmion ground states in magnetic metals. Nature.

[11] Münzer W (2010). Skyrmion lattice in the doped semiconductor Fe_1−x_Co_x_Si. Phys. Rev. B.

[12] Uchida M, Onose Y, Matsui Y, Tokura Y (2006). Real-space observation of helical spin order. Science.

[13] Heinze S (2011). Spontaneous atomic-scale magnetic skyrmion lattice in two dimensions. Nat. Phys..

[14] Emori S, Bauer U, Ahn S-M, Martinez E, Beach GSD (2013). Current-driven dynamics of chiral ferromagnetic domain walls. Nat. Mater..

[15] Ryu K-S, Thomas L, Yang S-H, Parkin S (2013). Chiral spin torque at magnetic domain walls. Nat. Nanotechnol..

[16] Kashid V (2014). Dzyaloshinskii-Moriya interaction and chiral magnetism in 3d−5d zigzag chains: tight-binding model and ab initio calculations. Phys. Rev. B.

[17] Yang H, Thiaville A, Rohart S, Fert A, Chshiev M (2015). Anatomy of Dzyaloshinskii-Moriya interaction at Co/Pt interfaces. Phys. Rev. Lett..

[18] Liu L (2012). Spin-torque switching with the giant spin Hall effect of tantalum. Science.

[19] Woo S, Mann M, Tan AJ, Caretta L, Beach GSD (2014). Enhanced spin-orbit torques in Pt/Co/Ta heterostructures. Appl. Phys. Lett..

[20] Woo S (2016). Observation of room-temperature magnetic skyrmions and their current-driven dynamics in ultrathin metallic ferromagnets. Nat. Mater..

[21] Fert A, Cros V, Sampaio J (2013). Skyrmions on the track. Nat. Nanotechnol..

[22] Jiang W (2015). Blowing magnetic skyrmion bubbles. Science.

[23] Avci CO (2019). Interface-driven chiral magnetism and current-driven domain walls in insulating magnetic garnets. Nat. Nanotechnol..

[24] Vélez S (2019). High-speed domain wall racetracks in a magnetic insulator. Nat. Commun..

[25] Wang H (2020). Chiral spin-wave velocities induced by all-garnet interfacial Dzyaloshinskii-Moriya Interaction in ultrathin yttrium iron garnet films. Phys. Rev. Lett..

[26] Ding, S. et al. Interfacial Dzyaloshinskii-Moriya interaction and chiral magnetic textures in a ferrimagnetic insulator. *Phys. Rev. B* **100**, 100406(R) (2019).

[27] Shao Q (2019). Topological Hall effect at above room temperature in heterostructures composed of a magnetic insulator and a heavy metal. Nat. Electron..

[28] Mccullian BA (2019). Spin-Hall topological Hall effect in highly tunable Pt/ferrimagnetic-insulator bilayers. Nano Lett..

[29] Quindeau A (2017). Tm_3_Fe_5_O_12_/Pt heterostructures with perpendicular magnetic anisotropy for spintronic applications. Adv. Electron. Mater..

[30] Shao Q (2018). Role of dimensional crossover on spin-orbit torque efficiency in magnetic insulator thin films. Nat. Commun..

[31] Suturin SM (2018). Role of gallium diffusion in the formation of a magnetically dead layer at the Y_3_Fe_5_O_12_/Gd_3_Ga_5_O_12_ epitaxial interface. Phys. Rev. Mater..

[32] Cooper JFK (2017). Unexpected structural and magnetic depth dependence of YIG thin films. Phys. Rev. B.

[33] Mitra A (2017). Interfacial origin of the magnetisation suppression of thin film yttrium iron garnet. Sci. Rep..

[34] Emori S (2014). Spin Hall torque magnetometry of Dzyaloshinskii domain walls. Phys. Rev. B.

[35] Tarasenko SV, Stankiewicz A, Tarasenko VV, Ferré J (1998). Bloch wall dynamics in ultrathin ferromagnetic films. J. Magn. Magn. Mater..

[36] Soumah L (2018). Ultra-low damping insulating magnetic thin films get perpendicular. Nat. Commun..

[37] Rosenberg ER (2018). Magnetism and spin transport in rare-earth-rich epitaxial terbium and europium iron garnet films. Phys. Rev. Mater..

[38] Fakhrul, T., Tazlaru, S., Beran, L., Zhang, Y., Veis, M., Ross, C.A. Magneto‐Optical Bi:YIG Films with High Figure of Merit for Nonreciprocal Photonics. *Adv. Op. Mat.* **7**, e1900056 (2019).

[39] Thiaville A, Rohart S, Jué É, Cros V, Fert A (2012). Dynamics of Dzyaloshinskii domain walls in ultrathin magnetic films. Europhys. Lett..

[40] Barla A (2016). Design and performance of BOREAS, the beamline for resonant X-ray absorption and scattering experiments at the ALBA synchrotron light source. J. Synchrotron Radiat..

[41] Vasili HB (2017). Direct observation of multivalent states and 4f → 3d charge transfer in Ce-doped yttrium iron garnet thin films. Phys. Rev. B.

[42] Singha A (2017). 4 f occupancy and magnetism of rare-earth atoms adsorbed on metal substrates. Phys. Rev. B.

[43] Teramura Y, Tanaka A, Thole BT, Jo T (1996). Effect of Coulomb interaction on the X-ray magnetic circular dichroism spin sum rule in rare earths. J. Phys. Soc. Jpn..

