# Supplementary material for: Interfacial Dzyaloshinskii-Moriya interaction arising from rare-earth orbital magnetism in insulating magnetic oxides
Source: Nat Commun. 2020 Feb 27;11:1090. doi: 10.1038/s41467-020-14924-7 (PMC7046643; doi:10.1038/s41467-020-14924-7)
Supplement: Supplementary file 1 — Supplementary Information [file 41467_2020_14924_MOESM1_ESM.pdf]

Supplementary Information:

Interfacial Dzyaloshinskii-Moriya Interaction Arising from Rare-Earth Orbital Magnetism in Insulating Magnetic Oxides

Caretta *et al.*

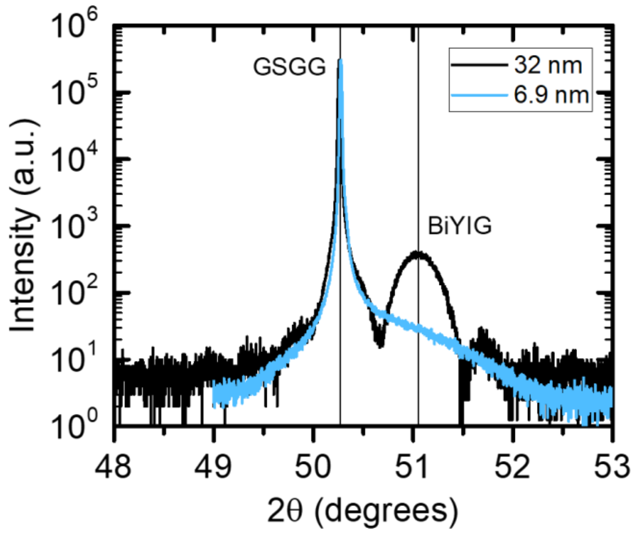

Supplementary Figure 1: X-ray diffraction of exemplary 6.9 nm and 32 nm GSGG/BiYIG samples. a.u., arbitrary units.

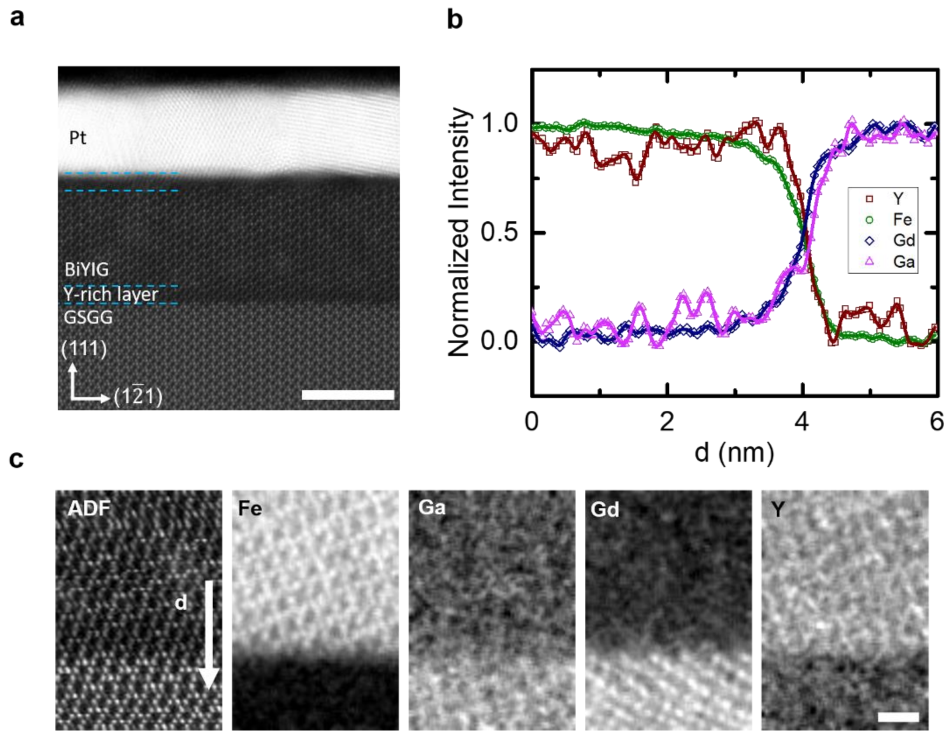

Supplementary Figure 2: Structural and Chemical Analysis a) Scanning transmission electron microscopy along the (10-1) direction of the GSGG/BiYIG(6.9 nm)/Pt(4 nm) sample. Scale bar: 5 nm. b) Electron energy loss spectroscopy (EELS) across the interface along the distance, d, shown in (c). c) Two-dimensional EELS maps for each elements, Fe, Ga, Gd and Y, as well as a simultaneously acquired annular dark-field (ADF) image. Scale bar: 1 nm.

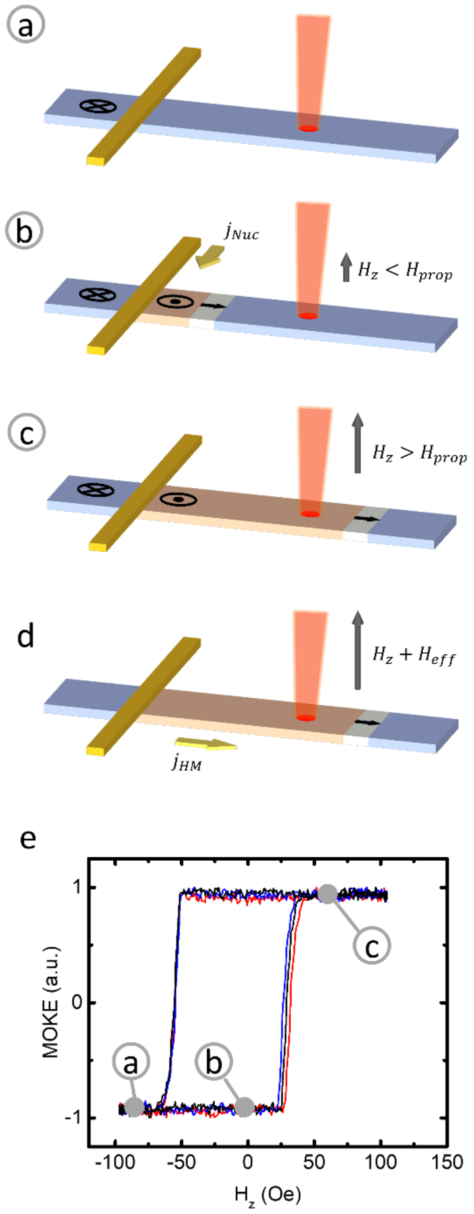

Supplementary Figure 3. Measuring the propagation field of a DW. a) Saturation state in a DW track with a Kerr laser downstream. b) Nucleated DW in the racetrack where the applied field H_z is less than the propagation field of the DW H_{prop}. c) DW passes under the laser spot when $H_z > H_{\mathrm{prop}}$. d) A current used in concert with H_z to drive a Néel DW. e) Resulting hysteresis loop from (a)-(d). a.u., arbitrary units.

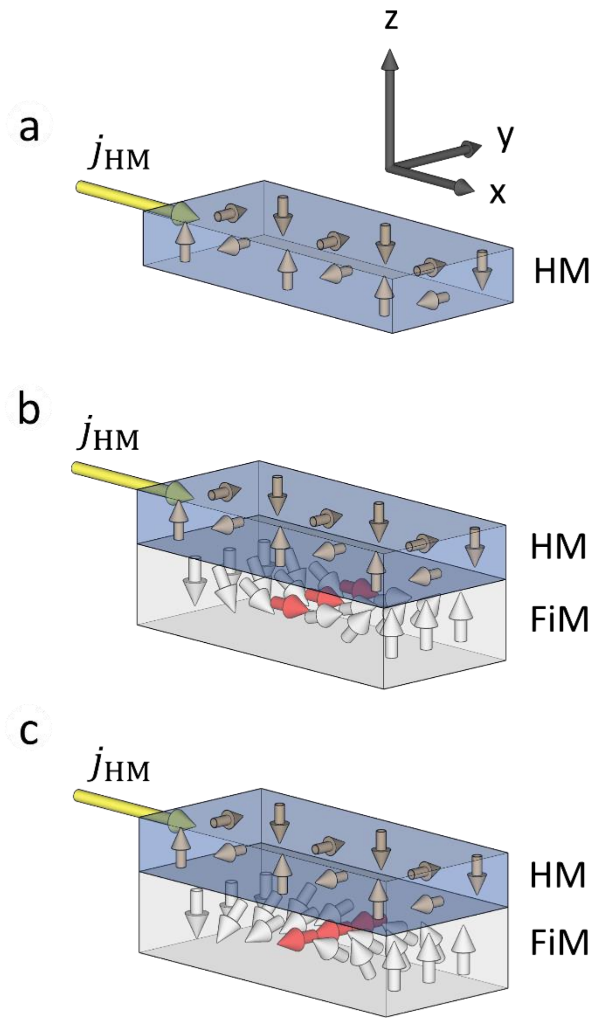

Supplementary Figure 4: Spin Hall effect acting on a Domain Wall. a) Spin Hall effect in heavy metal (HM) films converts a charge current j_{HM} into a transverse spin current. Arrows in heavy metal layer depict accumulated spin orientation at each interface. Spin accumulated at HM/FiM interfaces are along y-axis. HM film interfaced with a ferrimagnet (FiM) domain wall of b) Néel and c) Bloch orientation. Arrows in the FiM indicate net magnetisation orientation.

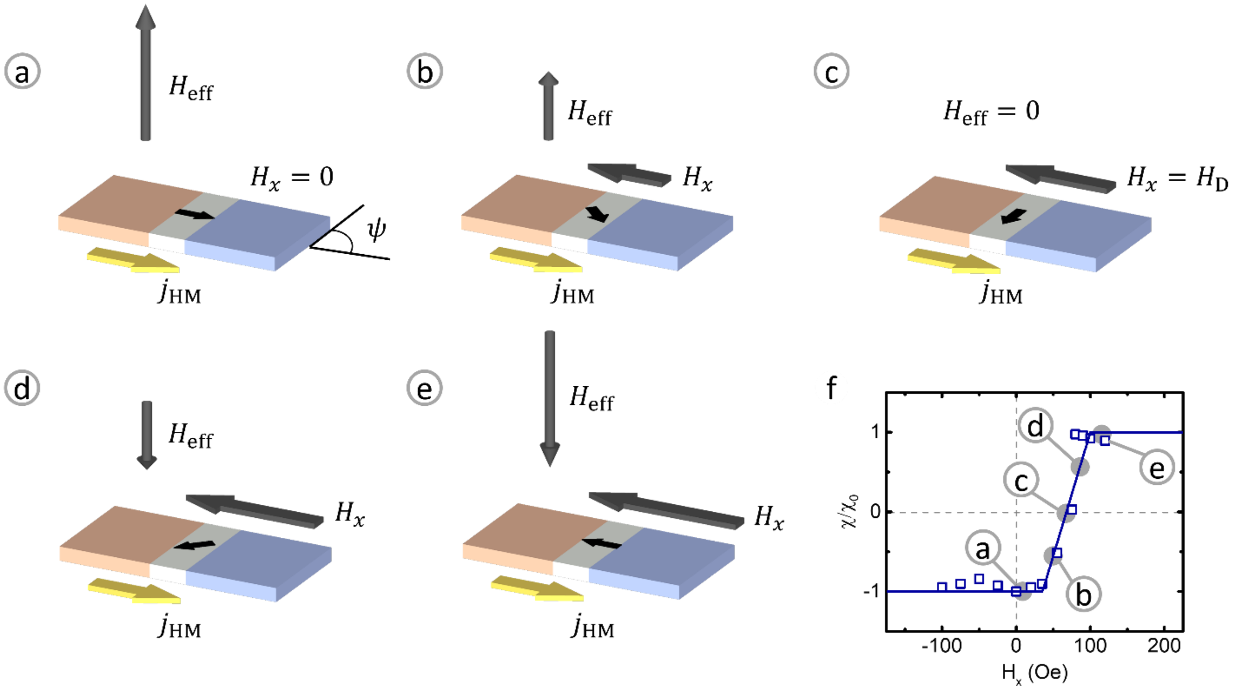

Supplementary Figure 5: **Measuring the DMI effective field H_D from spin Hall efficiency χ versus in-plane field H_x.** Effective magnetic field from a spin Hall current $H_{\rm eff}$ acting on a DMI-stabilized Néel DW when a) no in-plane field H_x is applied, b) small $H_x < H_D$, c) $H_x = H_D$, d) $H_x > H_D$ and e) when H_x is large enough to flip the chirality of the DW. f) Normalized Spin Hall efficiency χ/χ_0 as a function of in-plane field, and thus, DW orientation.

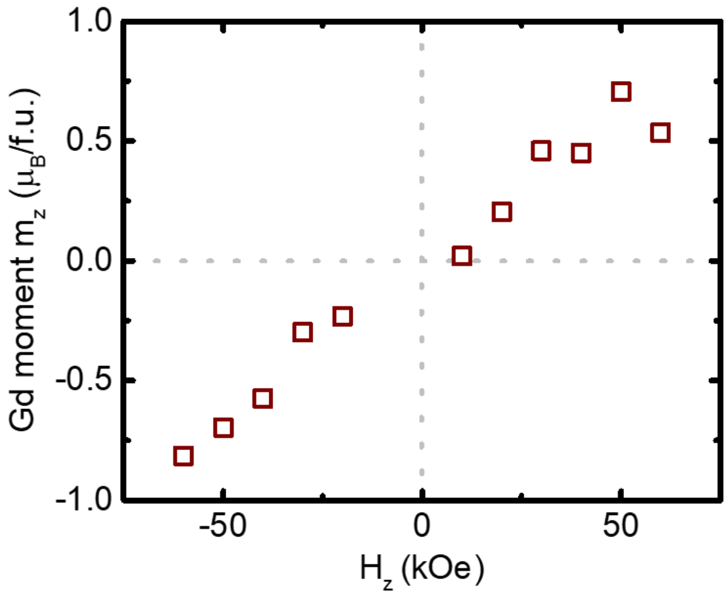

Supplementary Figure 6: Gd XMCD out-of-plane hysteresis loop. f.u., formula unit.

Supplementary Note 1: X-ray Diffraction and High-Resolution Scanning Transmission Electron Microscopy of BiYIG films

We characterise the structural quality of BiYIG films using x-ray diffraction (XRD), HAADF scanning transmission electron microscopy (STEM), and electron energy loss spectroscopy (EELS). Supplementary Figure 1 shows an XRD scan of 32 and 6.9 nm thick BiYIG grown on GSGG, revealing *(hhh)*-type reflections. Laue fringes around the 32 nm BiYIG peak demonstrate the high crystalline quality and thickness uniformity of the layer. Note that in the thinner 6.9 nm sample, fringes cannot be resolved. A high-resolution HAADF STEM image of the GSGG/BiYIG(6.9 nm)/Pt(4 nm) stack is depicted in Supplementary Figure 2a. The high-quality, epitaxial nature of the growth is clearly visible. The contrast in the STEM image indicates an off-stoichiometry interface region between the GSGG substrate and the BiYIG garnet. Complimentary line scan (Supplementary Figure 2b) and two-dimensional (Supplementary Figure 2c) EELS confirm that this $\sim 1\ nm$ thick interface region is comprised of Y-rich BiYIG with significant Gd and Ga intermixing from the substrate. Similar to the GGG/TmIG/Pt films, we anticipate that this off-stoichiometry layer to be a non-magnetic, dead layer. We also find a non-crystalline interface region between the BiYIG and sputtered Pt. This $\sim 1\ nm$ interface layer could be a result of high-energy sputter Pt deposition, and is also expected to be non-magnetic (dead) due to its non-crystalline nature.

Supplementary Note 2: Spin Hall motion of Néel domain walls stabilized by the DMI

In this section, we will describe the process used to measure the domain wall (DW) propagation field and the role of the DMI in spin-orbit torque motion of DWs.

Measuring the propagation field of a DW:

The substrate/garnet/Pt films are patterned into domain wall racetrack devices (Supplementary Figure 3a), where an orthogonally patterned Au stripline is used to nucleate DWs with an Oersted field. A laser is positioned down the track to sense the passing of a DW via the magneto-optical Kerr effect. We define the amount of magnetic field that is required to propagate a nucleated DW as the propagation field of the DW, H_{prop}. Measurement of the DW propagation field H_{prop} occurs through the following steps:

1) Using a magnetic field, the sample is initially saturated in the "down" magnetic state, as shown in Supplementary Figure 3a, and position "a" on the hysteresis loop in Supplementary Figure 3e. A current j_{Nuc} is passed through the orthogonal Au stripline to create a reversed domain and DW (denoted as white in Supplementary Figure 3b). This process occurs with no out-of-plane magnetic field applied (position "b" in Supplementary Figure 3e). If this sample has large enough DMI, then the DW will be Néel-type.

2) An out-of-plane magnetic field is ramped to drive the DW down the track. When the applied magnetic field H_z is lower than the propagation field H_{prop} of the DW, the DW will not move (Supplementary Figure 3b).

3) If $H_z > H_{\mathrm{prop}}$, then the DW will propagate down the track, passing over the laser spot Supplementary Figure 3c. This is denoted by position "c" in Supplementary Figure 3e, where the Kerr effect has changed sign. The zero-crossing of the Kerr effect signal is H_{prop}.

Measuring the spin Hall efficiency χ:

A charge current passing through a heavy metal with large spin-orbit coupling (ex – Ta, Pt, W) can be converted into a transverse spin current via the spin Hall effect, as shown in Supplementary Figure 4a, where spins of different orientation accumulate at different interfaces of the heavy metal[1,2]. By interfacing such heavy metals with the garnet films, the spin accumulation can induce a torque on the adjacent magnetic film DWs (Supplementary Figure 4b,c). The generated anti-damping-like torque from the spin Hall effect takes the form $\boldsymbol{\tau}_{\mathbf{AD}} \propto \mathbf{m} \times (\mathbf{m} \times \boldsymbol{\sigma}_{\mathbf{s}})$, where $\boldsymbol{\sigma}_{\mathbf{s}}$ is the spin accumulation polarization direction and $\mathbf{m}$ is the magnetisation of the DW moment. The spin Hall effective field, $\mathbf{H}_{\mathrm{SH}} \propto \mathbf{m} \times \boldsymbol{\sigma}_{\mathbf{s}}$, hence depends on the orientation of $\mathbf{m}$. In this geometry, charge current flows along the $\hat{\mathbf{x}}$ direction and spin polarization at the HM/magnetic interface occurs along the $\hat{\mathbf{y}}$ direction (in other words, $\boldsymbol{\sigma}_{\mathbf{s}}$ is along the y direction), as shown in Supplementary Figure 4b,c. The spin Hall effective field vanishes in Bloch DWs, since $\mathbf{m} = \pm\hat{\boldsymbol{y}}$ at the center of the wall, whereas it lies along $\hat{\mathbf{z}}$ (the out-of-plane easy axis) for Néel DWs, pointing in opposite directions for walls with internal moment $\mathbf{m} = +\hat{\mathbf{x}}$ and $\mathbf{m} = -\hat{\mathbf{x}}$. This is the basis of spin Hall torque magnetometry. since $\boldsymbol{\sigma}_{\mathbf{s}} = \hat{\mathbf{y}}$ is fixed, measuring the orientation of $\mathbf{H}_{\mathrm{SH}}$ provides the orientation of the DW moment. The DW must have some Néel component to feel the anti-damping-like torque from the spin Hall effect. Quantitatively, we can also write the effective field from the spin Hall effect as $H_{\mathrm{eff}} \equiv \chi\, j = \frac{\pi}{2}\chi_0\, j\, cos(\psi)$[3–5], where j is the current density, χ_0 is related to the spin Hall angle, ψ is the angle between the DW moment and the current flow direction. That is, when the DW is Bloch ($\cos(\psi) = 0$), $H_{\mathrm{eff}} = 0$ regardless of how much current is passed through the Pt overlayer. H_{eff} will only act on the DW if it has some Néel DW character ($|\cos(\psi)| > 0$).

Depending on the sign of the current flow through the Pt overlayer, H_{eff} can act to add-to or subtract-from the externally applied magnetic field H_z propagating a Néel DW (Supplementary Figure 3d), resulting in a change of the propagation field H_{prop} of the Néel DW. This is seen by the red and blue hysteresis loops in Supplementary Figure 3e and Main Text Figure 2b. Measuring the change in DW propagation field H_{prop} with current j_{HM} (Main Text Fig. 2c) is the spin Hall efficiency χ.

Measuring the strength of the DMI:

As a result of the interfacial DMI, homochiral Néel DWs form in materials that would otherwise form Bloch DWs[6,7]. And since the spin Hall torque only acts on Néel DWs, measuring the spin Hall efficiency χ can be an effective probe of the strength of the DMI in a sample. To do this, we measure χ as a function of in-plane magnetic field H_x. A sufficiently large in-plane field can reorient the moment of the DW, and hence influence the spin Hall efficiency χ[5]. The influence of H_x on the DW moment, and hence χ, is schematically shown in Supplementary Figure 5. Supplementary Figure 5a shows a fully Néel DW in a DMI film with no external applied in-plane magnetic field applied (H_x). A current passing through a Pt overlayer will produce an effective field H_{eff} on the DW proportional to $\cos(\psi) = -1$ for the Néel DW orientation shown in Supplementary Figure 5a. This results in a spin Hall efficiency $\chi/\chi_0 = -1$, as shown by letter "a" in the plot of χ/χ_0 versus H_x in Supplementary Figure 5f. If a small in-plane field H_x is applied opposing the orientation of the DW wall moment (Supplementary Figure 5b), the DW moment will begin to rotate from Néel to Bloch character, reducing $|\cos(\psi)|$ and hence the magnitude of H_{eff} andχ/χ_0 (Supplementary Figure 5f, letter "b"). If the strength of the applied H_x is strong enough to reorient the DW moment from Néel to Bloch (Supplementary Figure 5c), then H_{eff} will be reduced to zero (Supplementary Figure 5f, letter "c"). This in-plane field is equal to the DMI

effective field H_{D} and is used to quantify the DMI energy. Applying an even larger H_x will reorient the DW moment in the opposite direction, flipping the sign of H_{eff} (Supplementary Figure 5d) and χ/χ_0 (Supplementary Figure 5f, letter "d") until the DW moment has been completely flipped in the direction of the applied field H_x (Supplementary Figure 5e). In this final state, $\cos(\psi) = 1$, and χ/χ_0 is maximal. As a result, Supplementary Figure 5f can be used to directly read H_{D} from the zero-crossing of the curve.

Supplementary Note 3: Gd XMCD Hysteresis Loop

Element specific XMCD was performed on the GGG/TmIG(2.4 nm) film. We measure the magnetisation of Gd as a function of out-of-plane magnetic field H_z yielding a Gd hysteresis loop (Supplementary Figure 6). The response of the Gd is paramagnetic, implying that both substrate Gd and any interdiffused Gd from the GGG substrate into the TmIG dead layer is not ferrimagnetic at room temperature.

References:

1. Liu, L. *et al.* Spin-Torque Switching with the Giant Spin Hall Effect of Tantalum. *Science.* **336**, 555–558 (2012).
2. Pai, C.-F. *et al.* Spin transfer torque devices utilizing the giant spin Hall effect of tungsten. *Appl. Phys. Lett.* **101**, 122404 (2012).
3. Thiaville, A., Rohart, S., Jué, É., Cros, V. & Fert, A. Dynamics of Dzyaloshinskii domain walls in ultrathin magnetic films. *Europhys. Lett.* **100**, 57002 (2012).
4. Emori, S. *et al.* Spin Hall torque magnetometry of Dzyaloshinskii domain walls. *Phys. Rev. B* **90**, 4427 (2014).
5. Martinez, E., Emori, S., Perez, N., Torres, L. & Beach, G. S. D. Current-driven dynamics

of Dzyaloshinskii domain walls in the presence of in-plane fields: Full micromagnetic and one-dimensional analysis. *J. Appl. Phys.* **115**, 213909 (2014).

6. Emori, S., Bauer, U., Ahn, S.-M., Martinez, E. & Beach, G. S. D. Current-driven dynamics of chiral ferromagnetic domain walls. *Nat. Mater.* **12**, 611–616 (2013).
7. Ryu, K.-S., Thomas, L., Yang, S.-H. & Parkin, S. Chiral spin torque at magnetic domain walls. *Nat. Nanotechnol.* **8**, 527–533 (2013).
